# Risk of biochemical recurrence based on extent and location of positive surgical margins after robot-assisted laparoscopic radical prostatectomy

**DOI:** 10.1186/s12885-018-5229-1

**Published:** 2018-12-27

**Authors:** Marcq Gautier, Michelet Aude, Hannink Gerjon, Rizk Jerome, Sauvain Jean, Villers Arnauld, Saffarini Mo, H. Rochat Charles

**Affiliations:** 1 Urology Department, CHU Lille, F-59000 Lille, France; 2 University of Lille, GIVRE - MERCS - Module for Education and Research Collaboration in Statistics, F-59000 Lille, France; 3 ReSurg SA, chemin de la Vuarpillière 35, 1260 Nyon, Switzerland; 4 Orthopaedic Research Laboratory, Radboud University Medical Center, PO Box 9101, 6500HB, Nijmegen, The Netherlands; 5 Urology Department, Clinique Générale Beaulieu, 1204 Genève, Switzerland

**Keywords:** Prostate cancer, Prostatectomy, Robot-assisted, Laparoscopy, Biochemical recurrence, Positive surgical margins

## Abstract

**Background:**

There are no published studies on the simultaneous effect of extent and location of positive surgical margins (PSMs) on biochemical recurrence (BCR) after robot-assisted laparoscopic prostatectomy (RALP). The aim was to report the incidence, extent, and location of PSMs over the inclusion period as well as the rates of BCR and cancer-related mortality, and determine if BCR is associated with PSM extent and/or location.

**Methods:**

Retrospective review of 530 consecutive patients who underwent RALP between 2003 and 2012. Kaplan-Meier (KM) survival analyses and Cox regressions were performed to determine variables associated with BCR.

**Results:**

For the 530 operated patients, evaluated at a median of 92 months (IQR, 87–99), PSMs were observed in 156 (29%), of which 24% were focal. Out of 172 PSMs, 126 (73%) were focal and 46 (27%) were extensive. The KM survival using BCR as endpoint was 0.81 (CI, 0.78–0.85) at 5 years and was 0.67 (CI, 0.61–0.72) at 10 years; and using cancer-related mortality as endpoint was 0.99 (CI, 0.99–1.00) at 5 years and 0.95 (CI, 0.92–0.98) at 10 years. Multi-variable analysis revealed the strongest predictors of BCR to be Gleason score ≥ 8 (HR = 7.97; CI, 4.38–14.51) and 4 + 3 (HR = 3.88; CI, 2.12–7.07), lymph nodes invasion (HR = 3.42; CI, 1.70–6.91), pT stage 3b or 4 (HR = 3.07; CI, 1.93–4.90), and extensive apical PSMs (HR = 2.62; CI, 1.40–4.90) but not focal apical PSMs (HR = 0.86; CI, 0.49–1.50; *p* = 0.586).

**Conclusion:**

Extensive apical PSMs significantly increased the risk of BCR, independently from pT stage, Gleason score and lymph nodes invasion, while focal apical PSMs had no significant effect on BCR.

**Electronic supplementary material:**

The online version of this article (10.1186/s12885-018-5229-1) contains supplementary material, which is available to authorized users.

## Background

Positive surgical margins (PSMs) following radical prostatectomy are adverse outcomes, associated with the risk of biochemical recurrence (BCR) [1, 2]. Recent studies report a wide range of PSM incidence (12–39%) [3–8] which depends on tumor size, stage and localization, as well as surgeon experience [6, 9], and surgical approach [3, 10, 11].

The impact of PSM status on BCR is controversial: First, multifocal PSMs indicate that there is more cancer tissue left behind, but are not always associated with an increased risk of BCR [10–17]. Second, extensive PSMs increase the risk of BCR [2, 14, 15], even if the lack of consensus in reporting extent does not allow firm conclusions [1]. Third, apical and posterolateral PSMs are believed to present a greater risk of BCR than other locations [4, 5, 15, 18], though numerous studies found that location does not affect prognosis [4, 19]. Whatever their oncologic implications, PSMs often cause anxiety in affected patients, and could prompt adjuvant radiotherapy [2].

While some studies investigated the associations between BCR and PSM extent and location [4, 5], none considered their simultaneous effect. The authors therefore aimed to: (i) report the variations of incidence, extent, and location of PSMs following robot-assisted laparoscopic prostatectomy (RALP) over the inclusion period; (ii) report the rates of BCR and cancer-related mortality at 2, 5, and 10 years; and (iii) determine if BCR is associated with PSM extent and/or location independently from pathologic stage and Gleason score.

## Methods

### Study design

The authors retrospectively studied the records of all 530 consecutive patients (File1) that underwent RALP, between 2003 and 2012, all performed or supervised by the senior surgeon in a single center (CHR). The inclusion criteria were localized prostate cancer (cT1 to cT3). All patients provided written informed consent for their participation in this study. In addition to their routine follow-up (FU) visits, all patients were contacted to update their records and measure their prostate-specific antigen (PSA) serum levels, between 2015 and 2017. Unless confirmed to be deceased, patients who were not evaluated during this time interval were considered lost to follow-up (LTFU).

### Pre-operative evaluation

Pre-operative data included: age at diagnosis. PSA serum level, biopsy Gleason score and tumour clinical stage (cT) by rectal and imaging examinations (Table 1).

**Table 1:** Demographics, preoperative and pathological data

	Total		Evaluated		Lost to FU		*p*-value
	*n* = 530		c		*n* = 77		
*Follow-up (months) -* median (IQR)			92	(87–99)	47	(29–87)	
*Preoperative data*							
Age (years) - *median (IQR)*	63	(58–68)	63	(59–68)	61	(56–66)	*0.028*
Preoperative PSA - *median (IQR)*	6.4	(4.8–9.0	6.6	(4.9–9.0)	6.0	(4.7–9.2)	0.628
cT stage							*0.019*
*T1 (a,b,c)*	199	(38%)	175	(39%)	24	(31%)	
*T2a*	106	(20%)	82	(18%)	24	(31%)	
*T2b*	155	(29%)	136	(30%)	19	(25%)	
*T2c*	52	(10%)	48	(11%)	4	(5%)	
*T3*	11	(2%)	9	(2%)	2	(3%)	
*unknown*	7	(1%)	3	(1%)	4	(5%)	
D’Amico risk group							*0.005*
*low*	225	(42%)	205	(45%)	20	(26%)	
*intermediary*	196	(37%)	152	(34%)	44	(57%)	
*high*	97	(18%)	85	(19%)	12	(16%)	
*unknown*	12	(2%)	11	(2%)	1	(1%)	
Nerve sparing							0.471
*unilateral*	13	(2%)	12	(3%)	1	(1%)	
*bilateral*	498	(94%)	429	(95%)	69	(90%)	
*unknown*	19	(4%)	12	(3%)	7	(9%)	
*Pathological results*							
Pathological Gleason Score							*0.005*
≤ 6	214	(40%)	171	(38%)	43	(56%)	
3 + 4	178	(34%)	159	(35%)	19	(25%)	
4 + 3	73	(14%)	64	(14%)	9	(12%)	
≥ 8	58	(11%)	55	(12%)	3	(4%)	
*unknown*	7	(1%)	4	(1%)	3	(4%)	
pT stage							*0.004*
*pT2*	385	(73%)	321	(71%)	64	(83%)	
*pT3a*	80	(15%)	75	(17%)	5	(6%)	
*pT3b*	56	(11%)	52	(11%)	4	(5%)	
*pT4*	2	(0%)	2	(0%)	0	(0%)	
*unknown*	7	(2%)	3	(1%)	4	(5%)	
Involvment of lymph nodes	13	(2%)	12	(3%)	1	(1%)	0.483
Positive surgical margins							
Apical							
*focal*	53	(10%)	43	(9%)	10	(13%)	0.194
*extensive*	19	(4%)	19	(4%)	0	(0%)	0.054
Posterolateral							
*focal*	44	(8%)	39	(9%)	5	(6%)	0.604
*extensive*	20	(4%)	18	(4%)	2	(3%)	0.454
Base							
*focal*	26	(5%)	22	(5%)	4	(5%)	0.840
*extensive*	7	(1%)	7	(2%)	0	(0%)	na*
Bladder neck							
*focal*	3	(1%)	2	(0%)	1	(1%)	na*
*extensive*	0	(0%)	0	(0%)	0	(0%)	na*

*Not applicable, sample size too small

### Surgical technique

A 3-arm Da Vinci Surgical System (Intuitive Surgical, Sunnyvale, CA, USA) was used from 2003 to 2006 and was upgraded to a higher definition 4-arm system in January 2007 [20].

### Pathological evaluation

Prostate specimens were fixed in paraffin and prepared following the Stanford protocol [21]. Information collected included: pathologic stage of the tumour (pT), pathologic Gleason score, the presence of PSMs, defined as cancer glands observed at the inked surface of prostate specimens, and lymph nodes invasion (pN). The location of PSMs was defined as either apical, posterolateral, basal or at bladder neck. The extent of PSMs was noted as either focal (≤3 mm) or extensive (> 3 mm).

### Postoperative follow-up

For patients with undetectable PSA (< 0.1 ng/ml) at 6 weeks, PSA levels were measured every 6 months in the first 2 years, and once every year thereafter. BCR was defined by PSA > 0.2 ng/ml confirmed by two successive assays at any point during follow-up. None of the patients received adjuvant treatments, salvage treatments were only considered for patients with BCR.

### Statistical analysis

Categorical variables were described using numbers and percentages. Continuous variables were described using median, 95% confidence interval (CI) and inter-quartile range (IQR). Differences between patients evaluated after 2015 and those LTFU were assessed using Chi-2 tests or Fisher’s exact tests, where appropriate. Kaplan-Meier (KM) survival was estimated at 2, 5 and 10 years for two different endpoints: (i) BCR and (ii) cancer-related mortality. To account for competing risks, the cumulative incidence function was also used and revealed that the KM method exaggerated the incidence of BCR by only 0.8% at 10 years, which was deemed negligible. Uni- and multi-variable Cox regressions were performed, following the rule of a minimum of 10 events per variable (EPV) [22, 23],to determine associations of BCR with age, preoperative PSA, year of treatment, pathologic Gleason Score (≤6, 3 + 4, 4 + 3, ≥8), stage pT (pT2, pT3a, pT3b, pT4), lymph nodes invasion (none/ metastasized), as well as location and extent of PSM. Variables included in the final multi-variable model were identified by backward selection using the Akaike Information Criterion. Statistical analyses were performed using R version 3.4.1 (R Foundation, Vienna, Austria). The level of significance was set at *p* < 0.05.

## Results

**Fig. 1:**
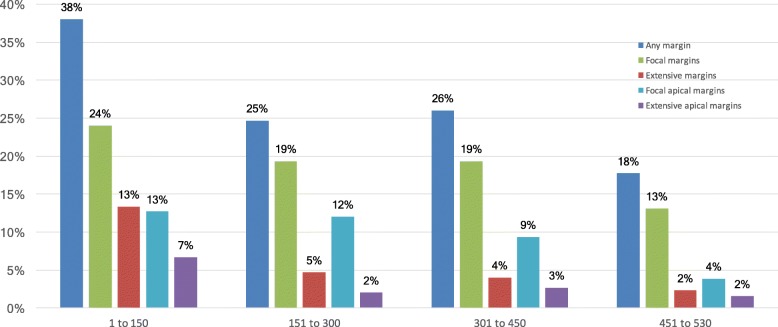
Incidence of posterior surgical margins (PSM) over the inclusion period

For the cohort of 530 patients operated, PSMs were observed in 156 (29%) patients, of which 18 (3%) had two or more PSMs (Additional file 1). Out of 172 PSMs, 126 (73%) were focal and 46 (27%) were extensive. The location of PSMs was apical in 72 (14%), posterolateral in 64 (12%), basal in 33 (6%), and at the bladder neck in 3 (1%) (Table 1). Patients at stage pT3a 57%) and pT3b/4 (72%) had a far greater incidence of PSMs than patients at stage pT2 (23%). The overall PSMs rates decreased from 38 to 18% over the inclusion period (Fig. 1). A total of 77 patients were lost to FU, leaving 453 for clinical evaluation at a median FU of 92 months (IQR, 87–99). Compared to patients evaluated, those LTFU comprised (Table 1) had similar proportion of high-risk tumors (16% vs. 19%; *p* = 0.504), and of extensive apical PSMs (0% vs 4%; *p* = 0.067), but a lower proportion of tumors at pT stage ≥3 (12% vs. 28%; *p* = 0.002).

**Fig. 2:**
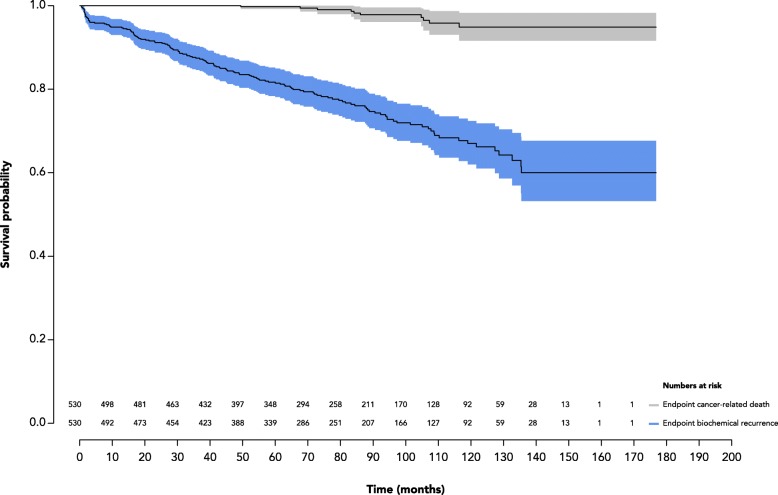
Kaplan-Meier survival curves for the initial cohort of 530 patients using two endpoints: (**a**) biochemical recurrence and (**b**) cancer-specific mortality

From the cohort of 453 patients evaluated, 138 (30%) had a BCR, of which 10 (2%) died of causes directly related to prostate cancer, and 9 (2%) died from unrelated causes. Compared to recurrence-free patients, patients who had BCR comprised (i) a higher proportion of pathological Gleason score ≥ 4 + 3 (49% vs. 17%; *p* < 0.001), (ii) a higher proportion of tumors at pT stage ≥3 (53% vs. 18%; *p* < 0.001), and (iii) a higher proportion of extensive PSMs (20% vs 5%; *p* < 0.001) (Table 2). Using BCR as endpoint, the KM survival at 2 years was 0.91 (CI, 0.88–0.93), at 5 years was 0.81 (CI, 0.78–0.85) and at 10 years was 0.67 (CI, 0.61–0.72). Using cancer-related mortality as endpoint, the KM survival at 2 years was 1.00 (CI, 1.00–1.00), at 5 years was 0.99 (CI, 0.99–1.00) and at 10 years was 0.95 (CI, 0.92–0.98) (Fig. 2).

**Table 2:** Comparison of patients with and without biochemical recurrence

	Recurrence-free *n* = 315	Biochemical Recurrence *n* = 138
Preoperative data		
Age – median (IQR)	63.0 (58.0–68.0)	64.0 (59.0–69.0)
Preoperative PSA – median (IQR)	6.0 (4.5–8.0)	8.0 (5.9–10.9)
Pathological results		
Pathological Gleason Score		
≤6	150 (48%)	21 (15%)
3 + 4	113 (36%)	46 (33%)
4 + 3	33 (10%)	31 (22%)
> 8	19 (6%)	36 (26%)
unknown	0 (0%)	3 (2%)
pT stage		
pT2	259 (82%)	62 (45%)
pT3a	40 (13%)	35 (25%)
pT3b	16 (5%)	36 (26%)
pT4	0 (0%)	2 (1%)
unkwon	0 (0%)	3 (2%)
Involvment of lymph nodes	2 (1%)	10 (7%)
Positive surgical margins		
Apical		
focal	29 (9%)	14 (10%)
extensive	6 (2%)	13 (9%)
Posterolateral		
focal	18 (6%)	21 (15%)
extensive	7 (2%)	11 (8%)
Base		
focal	11 (3%)	11 (8%)
extensive	4 (1%)	3 (2%)
Bladder neck		
focal	1 (0%)	1 (1%)
extensive	0 (0%)	0 (0%)

Univariable Cox regressions revealed BCR to be significantly associated with age at diagnosis (*p* = 0.015), preoperative PSA level (*p* < 0.001), year of treatment (p = 0.01); pathological Gleason score (p < 0.001), pT stage (p < 0.001), the lymph nodes invasion (p < 0.001) as well as the presence of focal posterolateral and base PSMs (respectively, p < 0.001 and *p* = 0.003) and extensive apical and posterolateral PSMs (respectively, *p* < 0.001 and *p* = 0.002) (Table 3). Multi-variable Cox regression revealed the strongest predictors of BCR to be Gleason score ≥ 8 (HR = 7.97; CI, 4.38–14.51; p < 0.001) and 4 + 3 (HR = 3.88; CI, 2.12–7.07; p < 0.001) compared to Gleason score ≤ 6, followed by lymph nodes invasion (HR = 3.42; CI, 1.70–6.91; p < 0.001), pT stage 3b or 4 (HR = 3.07; CI, 1.93–4.90; p < 0.001) compared to pT stage 2, and extensive apical PSMs (HR = 2.62; CI, 1.40–4.90; p = 0.003) but not focal apical PSMs (HR = 0.86; CI, 0.49–1.50; *p* = 0.586).

**Table 3:** Uni- and multi-variable Cox regressions of factors associated with biochemical recurrence

	Univariable			Multivariable		
	HR	95% CI	*p*-value	HR	95%	*p*-value
Age	1.03	(1.01–1.06)	0.015			
Preop PSA	1.02	(1.01–1.03)	< 0.001			
Year of treatment	1.10	(1.02–1018)	0.012			
Pathological Gleason Score						
≤6	REF			REF		
3 + 4	3.36	(2.00–5.64)	< 0.001	2.86	(1.68–4.88)	< 0.001
4 + 3	7.21	(4.13–12.60)	< 0.001	3.88	(2.12–7.07)	< 0.001
≥8	13.75	(7.96–23.74)	< 0.001	7.97	(4.38–14.51)	< 0.001
Stage pT						
pT2	REF			REF		
pT3a	3.12	(2.06–4.73)	< 0.001	2.28	(1.48–3.52)	< 0.001
pT3b & pT4^a^	7.37	(4.89–11.12)	< 0.001	3.07	(1.93–4.90)	< 0.001
Involvment of lymph nodes	8.84	(4.57–17.12)	< 0.001	3.42	(1.70–6.91)	< 0.001
Apical margins						
focal	1.10	(0.63–1.92)	0.742	0.86	(0.49–1.50)	0.586
extensive	3.80	(2.13–6.77)	< 0.001	2.62	(1.40–4.90)	0.003
Posterolateral margins						
focal	2.59	(1.62–4.14)	< 0.001			
extensive	2.73	(1.46–5.08)	0.002			
Base margins						
focal	2.60	(1.40–4.84)	0.003			
extensive^b^	1.45	(0.46 – 4.58)	0.525			
Neck margins						
focal	1.55	(0.21–10.98)	0.67			
extensive^c^						

^a^Only 2 patients were at stage pT4, both of which had biochemical recurrence

^b^Only 7 patients had extensive base margins, 3 of which had biochemical recurrence

^c^None of the patients had extensive neck margins

## Discussion

The principal finding of this study was that BCR was independently associated with extensive apical PSMs but not with focal apical PSMs, in addition to Gleason score (≥ 8 and 4 + 3), lymph nodes invasion, and pT stage (pT3b/pT4). This observation is important because the prostate apex is the most frequent PSM site for all radical prostatectomy approaches [24, 25]. There are several explanations as to why focal apical PSMs may be frequent but do not influence oncologic outcomes. First, the proximity of the urethral sphincter, neurovascular bundles and dorsal venous complex renders cancer excision at the apex most challenging for surgeons. Second, the variable configuration of the apex frequently causes iatrogenic intra-prostatic incisions, hypothetically leading to the creation of artefacts or ‘false’ PSMs [26]. Third, the sparse capsule and periprostatic tissue at the apex makes it difficult for histopathologists to distinguish intra-prostatic from extra-prostatic cancers [1].

Recent studies [27, 28] demonstrated that patients with PSMs have a higher risk of BCR and clinical progression but not necessarily of cancer-specific mortality. At a median follow-up of 92 months (IQR, 87–99), our BCR rate was 30% and cancer-specific mortality was 2%, both of which are within the range reported in recent studies [3–6, 8, 14, 16–18, 29]. At 10 years, our KM survival was respectively 67 and 89%, using BCR and cancer-specific death as endpoints, respectively.

It has been suggested that apical PSMs occur due to factors other than cancer aggressiveness, although their benign nature remains widely disputed [5, 30]. Our findings explain this controversy, which is likelydue to lack of distinction between focal and extensive PSMs at the apex. This further demonstrates the importance of adopting a standard method to report PSMs, including their precise extent and location [28]. The finding that focal apical PSMs are not associated with BCR is important, as it could help surgeons improve functional outcomes by sparing the sphincter, and could improve prognosis and decisions regarding additional radiotherapy (RT) [1, 2, 9]. The current rationale for administering local therapy is dichotomous: some advocate *adjuvant* RT immediately after surgery to all men with PSMs, others advocate *salvage* RT selectively to men if and when BCR is detected [2]. Our findings suggest that risk of BCR differs depending on PSMs location *and* extent, which should therefore be taken into account when considering adjuvant RT. In our series, multivariable analysis did not find extensive PSMs at other locations than the apex to be associated with BCR, likely due to the small number of observations in each subgroup. We recorded length of PSMs only as a categorical variable: focal (< 3 mm) or extensive (≥3 mm), [31] though the use of a continuous variable would have been more accurate and objective [32].

For the total cohort of 530 patients, PSMs were observed in 156 (29%) patients, of which 18 had two or more PSMs. The incidence of PSMs reported in recent radical prostatectomy series varies widely (12–35%) [3–6, 8, 14, 16–18, 29], partly due to the large inter-observer variability in identifying PSMs [28], and is also related to surgical experience [3, 6, 9, 12, 13]. For instance, in their study of 1701 RALPs, Sivaraman et al. [6] described a sharp decrease of PSM incidence from 25 to 20% after the first 350 patients. Sooriakumaran et al. [3] demonstrated continued improvements with increasing surgeon experience, estimating that over 1600 cases are required to decrease PSM rates below 10%. The observed rates of overall PSMs reduced over our inclusion period (Fig. 1). Extensive PSMs rates decreased drastically, immediately after the first 150 patients, while focal PSMs rates decreased more progressively, possibly due to their greater technical challenge.

The present study is limited by its small cohort size, which could influence our conclusions due to limited statistical power; we however respected the accepted rule of 10 events per variable [22, 23] for the Cox model. Although a non-negligible number of patients were lost to FU, they presented a less severe pathological profile (Table 1). Other limitations include lack of report of tumor volume, of Gleason score at PSM and of anterior margins, which were categorized as either basal, posterolateral or apical. Notwithstanding these limitations, the present study comprises a sizeable monocentric RALP series with adequate follow-up and account for surgical experience.

## Conclusion

Our study is the first to investigate the combined effects of PSM location and extent, after robotic assisted radical prostatectomy, and found that the risk of biochemical recurrence increased in the presence of extensive apical margins, independently of pathological Gleason score, pT stage, and lymph nodes invasion. In contrast, the presence of a focal apical margin did not increase the risk of biochemical recurrence. Our findings suggest that adjuvant radiotherapy should only be administered selectively depending on risk factors, including margin location and extent.

## Additional file

Additional file 1: Robotic Assisted Laparoscopic Prostatectomies (RALP) performed from 2003 to 2012. Database of the 530 patients treated by RALP from 2003 to 2012. (XLSX 103 kb)

## Abbreviations

BCR: BioChemical recurrence
CI: Confidence interval
cT: clinical stage of the tumor
FU: Follow-up
HR: Hazard ratio
IQR: InterQuartile range
KM: Kaplan-meier
LTFU: Lost to follow-up
PSM: Positive surgical margin
pT: Pathological stage of the tumor
RALP: Robot-assisted radical prostatectomy
