# Correction to: Risk of biochemical recurrence based on extent and location of positive surgical margins after robot-assisted laparoscopic radical prostatectomy

**DOI:** 10.1186/s12885-019-5296-y

**Published:** 2019-02-07

**Authors:** Gautier Marcq, Aude Michelet, Gerjon Hannink, Jerome Rizk, Jean Sauvain, Arnauld Villers, Mo Saffarini, Charles-Henry Rochat

**Affiliations:** 1 Urology Department, CHU Lille, F-59000 Lille, France; 2 GIVRE - MERCS - Module for Education and Research Collaboration in Statistics, University of Lille, F-59000 Lille, France; 3 ReSurg SA, Chemin de la Vuarpillière 35, 1260 Nyon, Switzerland; 4 Orthopaedic Research Laboratory, Radboud University, Medical Center, POBox 9101, Nijmegen, 6500HB The Netherlands; 5 Urology Department, Clinique Générale Beaulieu, 1204 Genève, Switzerland

**Correction to: BMC Cancer**

**https://doi.org/10.1186/s12885-018-5229-1**

Following publication of the original article [1], we have been notified that the authors’ last names have been incorrectly tagged as first names and vice-versa. The original publication has been corrected.

The correct author names are presented below:

Gautier MARCQ

Aude MICHELET

Gerjon HANNINK

Jerome RIZK

Jean SAUVAIN

Arnauld VILLERS

Mo SAFFARINI

Charles-Henry ROCHAT
